# Effects of a cognitive training program and sleep hygiene for executive functions and sleep quality in healthy elderly

**DOI:** 10.1590/1980-57642016dn11-010011

**Published:** 2017

**Authors:** Katie Moraes de Almondes, Maria Emanuela Matos Leonardo, Ana Maria Souza Moreira

**Affiliations:** 1 Associate Professor at the Department of Psychology and on the Postgraduate Program in Psychobiology, Federal University of Rio Grande do Norte, Natal RN, Brazil.; 2 Master's Student on the Postgraduate Program in Psychology, Federal University of Rio Grande do Norte, Natal RN, Brazil.; 3 Master's Degree on the Postgraduate Program in Psychology, Federal University of Rio Grande do Norte, Natal RN, Brazil.

**Keywords:** elderly, executive functions, sleep quality, cognitive training, sleep hygiene

## Abstract

**Introduction:**

The aging process causes changes in the sleep-wake cycle and cognition, especially executive functions. Interventions are required to minimize the impact of the losses caused by the aging process.

**Objective:**

To evaluate the effects of a cognitive training program and psychoeducation on sleep hygiene techniques for executive functions and sleep quality in healthy elderly.

**Methods:**

The participants were 41 healthy elderly randomized into four groups ([CG] control group, cognitive training group [CTG], sleep hygiene group [SHG] and cognitive training and hygiene group [THG]). The study was conducted in three stages:

1^st^ – assessment of cognition and sleep;
2^nd^ – specific intervention for each group;
3^rd^ – post-intervention assessment.

**Results:**

The results showed that the CTG had significant improvements in cognitive flexibility tasks, planning, verbal fluency and episodic memory, gains in sleep quality and decreased excessive daytime sleepiness. The SHG also had improved sleep quality, excessive daytime sleepiness and significant improvements in insights, planning, attention and episodic memory. The THG had significant gains in cognitive flexibility, problem solving, verbal fluency, attention and episodic memory.

**Conclusion:**

Cognitive training and sleep hygiene interventions were useful strategies for improving cognitive performance and sleep quality of healthy elderly, but there was no evidence that sessions combining cognitive training and psychoeducation on sleep hygiene enhanced the gains provided by these interventions applied individually.

## Abstract (pt)

**Introdução:**

O processo de envelhecimento provoca alterações no padrão de vigília sono dos idosos e na cognição, em especial no funcionamento executivo, sendo necessárias intervenções que visem minimizar os impactos das perdas ocorridas pelo processo de envelhecimento.

**Objetivo:**

Avaliar o efeito de um programa de treino cognitivo e das técnicas de higiene do sono para as funções executivas e qualidade de sono em idosos saudáveis.

**Métodos:**

Participaram da pesquisa 41 idosos saudáveis, divididos aleatoriamente em quatro grupos distintos (grupo controle [GC], grupo treino cognitivo [GTC], grupo higiene do sono [GHS] e grupo treino + higiene [GTH]). A pesquisa aconteceu em três etapas:

1ª – avaliação inicial da cognição e do sono;
2ª – intervenção específica destinada a cada grupo; e
3ª – reavaliação pós-intervenção.

**Resultados:**

Os resultados mostraram que o GTC apresentou melhora significativa em tarefas de flexibilidade cognitiva, planejamento, fluência verbal, memória episódica, qualidade de sono e diminuição da sonolência excessiva diurna. O GHS melhorou a qualidade do sono, sonolência diurna e teve melhoras significativas em capacidade de *insights*, planejamento, atenção e memória episódica. O GTH teve ganhos significativos na flexibilidade cognitiva, na resolução de problemas, na fluência verbal, na atenção e na memória episódica.

**Conclusão:**

Intervenções com treino cognitivo e higiene do sono são estratégias úteis na melhora do desempenho cognitivo e da qualidade de sono de idosos saudáveis, entretanto não houve evidências de que a intervenção com sessões combinando o treino cognitivo e psicoeducação da higiene do sono potencializem os ganhos proporcionados por essas intervenções isoladamente.

## INTRODUCTION

Prevalence studies in developing countries indicate that up to 37.7% of elderly have sleep-related complaints.^1^ The presence of complaints in relation to cognitive functioning is also common in this population, occurring with a prevalence of up to 50%.^2^

The aging process is accompanied by changes in the pattern of the sleep-wake cycle. Changes in duration (decreased total sleep time), efficiency (increased sleep latency, increased sleep fragmentation) and sleep architecture (reduced REM sleep time and slow wave sleep) are expected for healthy aging.^3,4^

Cognitive functioning is also impacted by the process of healthy aging. Among the cognitive skills that decline, executive functions (EFs), which include higher and basic cognitive processes for the formulation, execution, monitoring and correction of behaviors necessary to achieve goals and efficiently plan, are among the most impaired.^5^ Such impairments in executive functioning may negatively influence health, preservation of autonomy and quality of life.^5^

The difficulties encountered in carrying out executive tasks may be partly explained by changes in prefrontal areas (main substrate of AGs) and their connections (frontoparietal and frontosubcortical).^6^ In parallel, the sensitivity of the prefrontal regions to insufficient sleep is detrimental for the cognitive abilities relying on these brain areas.^7^

Recent studies have shown an association between poor sleep quality (and/or insufficient sleep) and worse cognitive performance, especially in attention and EFs.^8^ Therefore, interventions have been used to minimize such impairments, and psychoeducation on sleep hygiene techniques have been used to promote healthy sleep. This kind of intervention is positive for nocturnal sleep efficiency and reduced daytime naps.^9^

Similarly, cognitive training (CT) has been suggested as a possible strategy for improving or maintaining cognitive functioning. In the Brazilian context, research is still emerging on the effects of CT for executive functioning, but some studies have indicated positive effects of this type of intervention on the EFs and other cognitive processes.^10^

The aim of this study was to evaluate the effects of a cognitive training program and psychoeducation on sleep hygiene techniques for executive functions and sleep quality of healthy elderly.

## METHODS

**Participants.** Participants were recruited from a nonprofit civil entity. Exclusion criteria for all subjects were as follows:

[1] aged below 60;
[2] presence of visual or auditory uncorrected deficit;
[3] diagnosis of psychiatric disorders;
[4] signs of dementia or depression;
[5] physical or mental disability preventing completion of the proposed instruments;
[6] less than three years of education;
[7] diagnosis of a sleep disorder;
[8] absent from interventions more than once.

**Design and procedures.** Participants were randomized into blocks of four: Control Group (CG), Cognitive Training Group (CTG), Sleep Hygiene Group (SHG) and cognitive Training and Sleep Hygiene Group (THG). The randomization sequence was produced by random number generation on a computer. The research staff that performed the evaluations and carried out the interventions were blinded to the outcome measurements.

**Figure 1:**
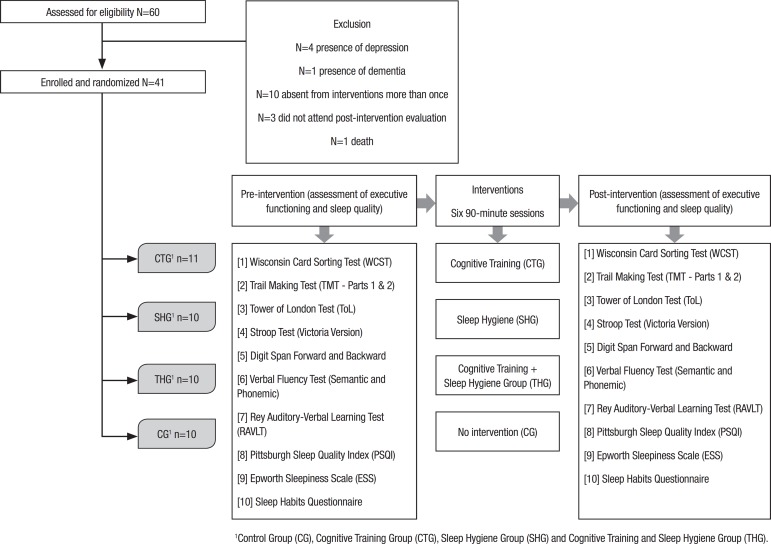
Flow chart of design and procedures.

Initial assessment was conducted on an individual basis (verification of inclusion criteria), followed by:

1^st^) assessment of executive functioning and sleep quality of the participants;
2^nd^) implementation of specific intervention for each group comprising six 90-minute sessions;
3^rd^) Revaluation of executive functions and sleep quality (Figure 1).

**Interventions.** *Cognitive training.* The activities were carried out to promote the skills of planning, ordering, attention, working memory, problem solving, verbal fluency and mental flexibility. This cognitive training program was based on the studies of Irigaray, Gomes Filho and Schneider^11^ and Lima-Silva et al.^12^

*Sleep hygiene.* These are a series of behavioral and environmental recommendations to promote healthy sleep through the gradual restructuring of poor habits in relation to sleep. Stimulating questions were asked and conducted on the following topics: importance of sleep, sleep and aging, sleep and executive functions, sleep environment and habits that influence sleep.

**Measures.** Initial assessment for study inclusion:

[1] Mini-Mental State Examination (MMSE)^13^ (used for cognitive impairment screening);
[2]Geriatric Depression Scale (GDS-15);^14^
[3] Pfeffer's Functional Activities Questionnaire^15^ (assesses functioning based on degree of dependence for performing instrumental activities of daily living).^15^

For assessment of cognitive functioning and sleep:

[1] Wisconsin Card Sorting Test (WCST)^16^ – version for elderly (evaluates executive functions);
[2] Trail Making Test (TMT – Parts 1 & 2)^17^ (evaluates speed of processing, mental flexibility and executive functioning);
[3] Tower of London Test (ToL)^18^ (assesses planning abilities and problem solving);
[4] Stroop Test (Victoria Version)^19^ (assesses selective attention and inhibitory control);
[5] Digit Span Forward and Backward – WAIS^20^ (measures working memory's number storage capacity;
[6] Verbal Fluency Test (Semantic and Phonemic):^21^ evaluates semantic fluency using animals categories and phonological fluency test using the FAS;
[7] Rey Auditory-Verbal Learning Test (RAVLT),^22^ used in evaluation of declarative memory;
[8] Pittsburgh Sleep Quality Index (PSQI):^23^ used to assess sleep quality;
[9] Epworth Sleepiness Scale (ESS);^24^
[10] Sleep Habits Questionnaire^25^ (assesses sleep habits and sleep conditions).

**Ethics procedures.** This study was approved by the Research Ethics Committee of the Federal University of Rio Grande do Norte with the CAAE registration number: 19114413.9.0000.553.

**Statistical analysis.** Data analysis was performed using the SPSS 20.0 Program (Statistical Package for the Social Sciences), assigning a 5% significance level for all statistical tests. Inferential and descriptive statistics were used for data evaluation. The Chi-square test (χ^2^), generalized Fisher test and Kruskal-Wallis test (H) were employed for comparisons between groups regarding absolute frequency of the variables: demographic data, MMSE, PFAQ and GDS. The Wilcoxon test (T) and McNemar test were used for intragroup comparisons, whereas the Kruskal-Wallis and Chi-square tests were applied for intergroup comparisons. The Mann-Whitney test (U) was used for pairwise comparisons of the same variables. Also, percentage delta differences were calculated (post-test score minus the pre-test score) for numeric variables.

## RESULTS

**Background measures.** The sample of 41 participants comprised 10 elderly subjects in the control group (CG), 11 in the cognitive training group (CTG), 10 in the Sleep Hygiene Group (SHG) and 10 in the cognitive Training and sleep Hygiene Group (THG). These groups were compared in terms of sociodemographic variables, depressive symptoms, functioning and dementia screening (Table 1).

**Table 1:** Sociodemographic characteristics and screening for cognitive impairment, depressive symptoms and functioning.

Group Variable	CG (n = 10)	CTG (n = 11)	SHG (n = 10)	THG (n = 10)	P
**Gender**	**n (%)**	**n (%)**	**n (%)**	**n (%)**	0.27*
Female	7 (70)	10 (90.91)	10 (100)	8 (80)	
Male	3 (30)	1 (9.09)	0 (0)	2 (20)	
**Age (years)**	**Mean (SD)**	**Mean (SD)**	**Mean (SD)**	**Mean (SD)**	0.131**
	66.9 (7.52)	72.2 (8.58)	66.4 (7.427)	72.0 (7.09)	
**Marital Status**	**n (%)**	**n (%)**	**n (%)**	**n (%)**	0.27*
Single	1 (10)	3 (27.27)	0 (0)	2 (20)	
Married	6 (60)	2 (18.18)	4 (40)	3 (30)	
Divorced	1 (10)	1 (9.09)	3 (30)	0 (0)	
Widowed	2 (20)	5 (45.45)	3 (30)	5 (50)	
**Education**	**n (%)**	**n (%)**	**n (%)**	**n (%)**	0.051**
Read and write	0 (0)	2 (18,18)	5 (50)	2 (20)	
Primary school	3 (30)	2 (18,18)	2 (20)	4 (40)	
High school	4 (40)	6 (54.54)	3 (30)	4 (40)	
College	3 (30)	1 (9.090)	0 (0)	0 (0)	
**Retirement**	**n (%)**	**n (%)**	**n (%)**	**n (%)**	0.15*
Yes	9 (90)	6 (54.55)	5 (50)	8 (80)	
No	1 (10)	5 (45.45)	5 (50)	2 (20)	
Incomes***	**n (%)**	**n (%)**	**n (%)**	**n (%)**	0.102**
< US$ 246.5	3 (30%)	5 (45.5%)	5 (50%)	4 (40%)	
>US$ 246.5 and US$ 492.5	0 (0%)	3 (27.3%)	4 (40%)	2 (20%)	
> US$ 492.5 and US$ 739	1 (10%)	2 (18.2%)	1 (10%)	2 (20%)	
> US$ 739 and US$ 1232.5	2 (20%)	1 (9.1%)	0 (0%)	1 (10%)	
>US$ 1232.5 and US$ 1725.5	2 (20%)	0 (0%)	0 (0%)	1 (10%)	
>US$ 1725.5	2(20%)	0 (0%)	0 (0%)	0 (0%)	
MMSE	**Mean (SD)**	**Mean (SD)**	**Mean (SD)**	**Mean (SD)**	
	28 (2.66)	28 (1.86)	28.2 (1.54)	28.5 (2.59)	0.745**
**PFAQ**	**Mean (SD)**	**Mean (SD)**	**Mean (SD)**	**Mean (SD)**	
	1 (1.11)	0.4 (0.82)	1 (1.05)	0.7 (0.94)	0.517**
**GDS-15**	**Mean (SD)**	**Mean (SD)**	**Mean (SD)**	**Mean (SD)**	
	2.4 (1.26)	1.5 (1.12)	2.7 (1.41)	1.5 (0.85)	0.080**

* p value refers to Chi-square test and generalized Fisher test. Note.

** p refers to Kruskal Wallis test. Note.

*** incomes converted into US dollars. MMSE: Mini-Mental State Examination; PFAQ: Pfeffer's Functional Activities Questionnaire; GDS - 15: Geriatric Depression Scale.

## Pre-intervention Analysis (CG, CTG, SHG and THG)

*Sleep measures.* There were no significant differences between groups for the sleep quality parameter (H (3)=2.17; p=0.24) or excessive daytime sleepiness (H (3)=3.98; p=0.26). All groups had poor sleep quality (PSQI>5) and only the SHG had excessive daytime sleepiness (ESS>10).

*Cognitive measures.* There was a statistically significant difference between groups in the scores on the ToL (H (3)=11.2, p=0.011) and Digit Span Forward (H (3)=9.19, p=0.027) tests. For the ToL variable, the differences were detected at the intersection of the CG×CTG (U=21; p=0.016), CG×SHG (U=15; p=0.007) and CG×THG (U=10; p=0.002) groups, suggesting that the CG had significantly higher performance in planning and problem-solving skills. The same was observed on the Digit Span Forward, with significant differences found at the intersection of the CTG×CG (U=24, 5, p=0.029), CG×SHG (U=15; p=0.07) and CG×THG (U=19; p=0.016) groups, indicating that the CG had significantly better performance on this short-term memory task.

*Intragroup comparison analysis – sleep and measures.* In the intervention groups, there was a significant improvement in sleep quality and a reduction in excessive daytime sleepiness complaints (Table 2), but no significant changes in sleep habits were observed in the elderly.

**Table 2:** Intragroup comparison analysis* - Sleep and cognitive outcomes.

Variable	CG				CTG				SHG				THG		
	Pre Mdn	Post Mdn	P		Pre Mdn	Post Mdn	P		Pre Mdn	Post Mdn	P		Pre Mdn	Post Mdn	P
**Executive**															
WCST - Perseverative errors	30.5	21.5	0.332		32.0	21.0	0.036		37.0	16.5	0.066		23.0	14.5	0.025
WCST - Failure to maintain set	0.0	1.0	0.142		1.0	1.0	0.723		1.0	1.0	1.00		1.0	2.0	0.317
WCST - Categories generated	3.8	4.0	0.414		3.0	5.0	0.017		3.0	5.0	0.021		3.5	5.5	0.141
WCST - Conceptual Level Responses	59.0	61.0	0.285		57.0	69.0	0.062		50.5	68.0	0.037		56.5	64.5	0.066
Digit Span Backward	4.0	4.5	0.762		4.0	4.0	0.180		2.5	4.0	0.130		4.0	4.5	0.327
Stroop Test 3^rd^ card - time	0:00:32	0:00:35	0.574		0:00:35	0:00:32	0.305		0:00:49	0:00:43	1.00		0:00:35	0:00:31	0.092
Stroop Test 3^rd^ card - errors	1.5	1.5	0.552		3.0	2.0	0.078		4.0	2.0	0.291		2.5	1.5	0.351
TMT - Part B	146.0	163.5	0.906		185	201	0.197		194.5	255.5	0.959		174	165.5	0.799
TMT - Measure interference	1.25	1.2	0.202		1.0	1.1	0.303		1.3	2.1	0.037		0.60	1.0	0.278
ToL	31.5	28.0	0.008		26.0	30.0	0.011		27.0	30.5	0.007		26.5	30.0	0.012
Verbal Fluency Test - Phonemic	28.0	33.5	0.074		41.0	43.0	0.029		22.0	31.5	0.109		33.0	40.5	0.024
**Attention**															
Stroop Test 1^st^ card - time	0:00:17	0:00:18	0.570		0:00:19	0:00:18	0.306		0:00:20	0:00:21	0.256		0:00:19	0:00:19	0.052
Stroop Test 1^st^ card- errors	0.00	0.00	1.00		0.0	0.0	0.564		0.0	0.0	0.317		0.0	0.0	1.00
Stroop Test 2^nd^ card -time	0:00:24	0:00:20	0.282		0:00:28	0:00:24	0.154		0:00:26	0:00:26	0.035		0:00:34	0:00:24	0.017
Stroop Test 2^nd^ card - errors	0.00	0.00	0.102		0.0	0.0	0.187		0.0	0.0	0.180		0.0	0.0	0.157
TMT - Part A	70.5	68.5	0.074		70.0	89.0	0.657		88.5	79.5	0.059		83.5	79.5	0.201
**Memory**															
Verbal Fluency Test (Semantic)	28	33.5	0.758		41.0	43.0	0.682		22	31.5	0.944		33.0	40.5	0.005
Digit Span Forward	8.0	6.0	0.066		5.0	6.0	0.860		6.0	5.5	0.518		6.0	6.0	1.00
RAVLT - Learning	42.5	49.0	0.005		39.0	51.0	0.038		41.0	49.0	0.005		41.5	50.0	0.011
RAVLT - A6	9.0	9.5	0.259		9.0	11.0	0.046		6.5	10.5	0.030		9.0	12.0	0.008
RAVLT - A7	8.5	11.5	0.015		9.0	10.0	0.159		7.5	10.0	0.011		9.0	12.0	0.043
RAVLT - Recognition	11.0	12.5	0.301		9.00	13.00	0.223		6.5	11.0	0.045		11.0	13.0	0.009
**Sleep**															
PSQI	7.0	5.5	0.255		6.0	5.0	0.011		7.5	4.0	0.005		5.0	3.5	0.020
ESS	6.5	10.0	0.078		9.0	3.0	0.018		11.0	6.0	0.008		9.0	6.0	0.010

* p value refers to Wilcoxon test. WCST: Wisconsin Card Sorting Test; TMT: Trail Making Test; ToL: Tower of London Test; RAVLT: Rey-Auditory Verbal Learning Test; PSQI: Pittsburgh Sleep Quality Index; ESS: Epworth Sleepiness Scale.

## Intragroup comparison analysis – Cognitive measures

*Cognitive Training Group (CTG).* The intervention with CT promoted improved executive functioning, specifically in cognitive flexibility skills, planning, problem-solving and verbal fluency; as well as in mnemonic capacity, especially for learning and short-term memory, evidenced by significant differences in WCST- perseverative errors, WCST- number of completed categories, ToL, Fluency (FAS), RAVLT- Learning and RAVLT-A^6^. Finally, the gains in attentional capacity of the elderly post intervention were modest, indicating an absence of any major benefits from the cognitive training sessions for attention (Table 2).

*Sleep Hygiene Group (SHG).* The elderly from the SHG group, according to WCST analysis, performed better on the number of completed categories and conceptual level responses, indicating the techniques in sleep hygiene may have resulted in gains for the executive functions, more specifically insights capacity, cognitive flexibility, planning and problem solving, as well as selective attention ability and episodic memory (Table 2).

*Cognitive Training and Sleep Hygiene Group (THG).* There was statistical significance for the variables WCST - perseverative errors, ToL, FAS, Stroop second card - time, Fluency (animals), RAVLT-Learning, RAVLT-A^6^, RAVLT-A^7^ and RAVLT-recognition (Table 2). These results indicate that the combined intervention resulted in gains for some components of executive functions, such as mental flexibility, planning and verbal fluency. In addition, significant improvements on almost all of the episodic memory tests were evident, indicating gains in mnemonic capacity for a possible transfer of executive functions training, as well as sleep hygiene.

*Control Group (CG).* The CG elderly worsened significantly in planning ability and problem solving, but improved in episodic memory, especially delayed recall (Table 2).

*Comparison between interventions - Sleep Measures.* The analysis of the deltas groups revealed significant differences in the sleep quality parameter and excessive daytime sleepiness (Table 3). Concerning sleep quality, differences were found between the SHG (Md=7.4)×CTG (Md=14.27) (U=19.0, p=0.01) and SHG (Md=7.4)×CG (Md=13.95) (U=15.5; p=0.007), indicating that the gains acquired by the SHG were statistically superior to the gains acquired by the CTG and CG. With regard to the excessive daytime sleepiness, the differences were between CG×CTG (U=14.5, p=0.004), CG×SHG (U=7.0, p=0.01) and CG×THG (U=7.0, p=0.00), indicating that the CG had a significantly negative effect for daytime sleepiness when compared to the other experimental groups . This result indicates that all groups submitted to interventions had significant improvements in sleepiness complaints, while the CG worsened (Table 3).

**Table 3:** Comparison between interventions* - Sleep and cognitive outcomes.

Variable	CG	CTG	SHG	THG	P
**Executive**	**Delta**	**Delta**	**Delta**	**Delta**	
WCST - Perseverative errors	-5.4	-6.9	-12.6	-11.4	0.405
WCST - Failure to maintain set	1.0	-0.2	-0.2	0.3	0.696
WCST - Categories generated	0.2	1.2	1.9	1.3	0.051
WCST - Conceptual Level Responses	7.3	11.1	15.3	15.5	0.807
Digit Span Backward	0.2	-0.3	0.8	-0.5	0.181
Stroop Test 3^rd^ card - time	0:00:00	-0:00:01	0:00:00	-0:00:12	0.887
Stroop Test 3^rd^ card - errors	1.7	-1.8	-1.1	-0.9	0.375
TMT - Part B	-2.8	-17.4	2.4	-2.7	0.775
TMT - Measure interference	0.3	-0.3	0.8	0.4	0.075
ToL	-3.2	3.8	3.9	2.6	0.000
Verbal Fluency Test - Phonemic	5.7	4.8	4.1	7.7	0.700
**Attention**	**Delta**	**Delta**	**Delta**	**Delta**	
Stroop Test 1st card - time	0:00:00	0:00:00	0:00:01	-0:00:02	0.121
Stroop Test 1st card- errors	-0.6	0.1	-0.2	-0.3	0.927
Stroop Test 2nd card -time	-0:00:02	-0:00:02	-0:00:03	-0:00:05	0.772
Stroop Test 2nd card - errors	-0.7	-0.1	-0.3	-0.2	0.828
TMT - Part A	-8.9	-2.9	-35	-17.7	0.471
**Memory**	**Delta**	**Delta**	**Delta**	**Delta**	
Verbal Fluency Test (Semantic)	0.0	-0.5	0.1	3.9	0.028
Digit Span Forward	-1.2	-0.1	0.3	0.0	0.128
RAVLT - Learning	10.2	5.6	9.8	6.6	0.283
RAVLT - A6	0.9	4.4	3.1	3.7	0.097
RAVLT - A7	2.6	1.2	2.5	2.0	0.522
RAVLT - Recognition	0.5	1.3	3.5	5.0	0.127
**Sleep**	**Delta**	**Delta**	**Delta**	**Delta**	
PSQI	-0.9	-1.5	-4.1	-1.9	0.023
ESS	2.5	-4.6	-4.3	-3.9	0.002

* p value refers to Kruskal-Wallis test. WCST: Wisconsin Card Sorting Test; TMT: Trail Making Test; ToL: Tower of London Test; RAVLT: Rey-Auditory Verbal Learning Test; PSQI: Pittsburgh Sleep Quality Index; ESS: Epworth Sleepiness Scale.

*Comparison between interventions - Cognitive Measures.* The analysis of delta revealed statistically significant difference in the ToL and Verbal Fluency task (animals) between the groups pre and post intervention (Table 3). For the ToL variable, the differences were between the CG×CTG groups (U=107.0, p=0.0013), CG×SHG (U=99.5, p=0.0012) and CG×THG (U=97.5, p=0.0014) indicating that the CG had significantly negative effect for planning ability and problem solving when compared to the experimental groups. This result indicates that all groups submitted to interventions had significant improvements in this skill, while the CG worsened significantly.

On the Verbal Fluency (animals) task, significant differences were found at the intersection of THG×CG (U=85.0, p=0.043) and THG×SHG (U=88.5, p=0.022) groups, indicating that THG had the greatest gains on this verbal fluency task, used in this study as a measure of semantic memory.

## DISCUSSION

The aim of this study was to evaluate and compare the effects of cognitive training (CT) and psychoeducation on sleep hygiene for executive functions and sleep quality of healthy elderly.

Concerning the pre-intervention analysis, there was homogeneity of the different groups in relation to demographic data, sleep and cognitive variables. The post-intervention intragroup comparisons showed that the CT intervention for executive functions improved the cognitive performance of the healthy elderly, not only on the skills directly trained in the sessions, but also on other functions.

Cognitive training is well documented as a strategy to provide benefits for cognitive functioning of the elderly through practical skills training, with adaptable difficulty levels, and both stimulating and rewarding environments. Some studies suggest that the effects on cognitive performance can occur in a particular spectrum – the target cognitive domain of the intervention, e.g. executive functions - or on a broader level – with transfer to other areas beyond the focus of the intervention.^26^

In this study, both of the groups submitted to CT, when compared before and after intervention, showed significant improvements in cognitive flexibility, planning, problem solving, phonological verbal fluency and episodic memory. These significant gains were also found in the studies of Irigaray et al.^11^ and Wang et al.,^27^ which also involved cognitive training sessions for executive functions in healthy older adults.

The mechanisms by which these gains occur are not yet clear, though some researchers emphasize brain neuroplasticity as a key to the maintenance of CT gains and the transfer process.^28^ Engvig et al.^29^ identified a positive relationship between better mnemonic performance and macro-structural changes in the brains of elderly patients undergoing CT, indicating that this type of intervention can influence the mechanisms of brain changes at a structural level in senescence.

Another hypothesis holds that cognitive flexibility and verbal fluency are functions less susceptible to the effects of aging. Park, Gutchess, Meade & Stine-Morrow^30^ propose it is easier training activities that involve more "intact" functions, since the level of automation of these processes involves less cognitive effort. Thus, this might explain the gains found in verbal fluency and cognitive flexibility after training sessions, findings similar to the study of Lira et al.^10^ and Silva et al.^31^

Regarding sleep variables, even in the post-intervention intragroup analysis, no change in sleep patterns was found for any of the groups, probably due to the use of a dichotomous instrument for the evaluation of this variable, limiting the possibilities for statistical research on this parameter. In addition, the CT yielded gains not only for cognitive performance of elderly people undergoing this intervention, but also improved sleep quality and decreased excessive daytime sleepiness complaints. Also in relation to intra-group post-intervention analysis, the only group that participated in the sessions of psychoeducation on sleep hygiene techniques (SHG), showed significant improvement in sleep quality, excessive daytime sleepiness, EFs, selective attention and episodic memory.

Evidence indicates that sleep and cognitive abilities are affected by similar processes that occur in the brain due to aging, such as atrophy, synaptic degeneration, reduced blood flow and other neurochemical changes. Assuming that cognitive training can promote neural plasticity, it follows that the activities carried out in the training sessions could improve sleep by reducing the impact of these processes, most likely through synaptic plasticity promoted by the training.^32^

In addition, cognitive training has a beneficial effect on sleep architecture, which can improve overall sleep quality, as was seen in the CTG. During aging, there are often changes in sleep architecture (decrease in slow waves and delta), referred to as key mechanisms for recovery of the prefrontal cortex. In this sense, the cognitive training can act as a catalyst, "forcing" the brain to have more episodes of slow wave sleep and REM sleep and thus be able to process the acquired learning, changing sleep architecture.^33^ In conclusion, one can consider that the post-intervention improvement in the indices of sleep quality and daytime sleepiness observed in the groups submitted to cognitive training may have been caused by changes in sleep architecture, which increased the REM and slow wave stage, allowing the elderly to have more nights of restorative sleep and report better sleep quality and less daytime sleepiness. This increase may have improved cognitive performance in the case of the SHG.

Concerning the intervention of psychoeducation on sleep hygiene, analysis of deltas showed that the SHG and THG were the groups with the most improved sleep. The majority of studies addressing sleep hygiene have obtained modest results.^34^ However, some significant results can be explained by the low adherence of the elderly to the intervention, whereby the techniques taught or the meetings do not motivate them in a satisfactory manner, requiring the creation of motivational strategies (encouraging the formation of relationships, homework) that promote incorporation of the proposed changes into participants´ routines.^35^

Finally, regarding the combined interventions (THG), significant improvements in sleep quality, sleepiness as well as in the executive component and memory (episodic and semantic) were observed on analysis of the deltas. Consequently. it can be observed that the THG had similar gains to the SHG on transfer measures, possibly indicating that psychoeducation gains for sleep have relevance in these cognitive functions.^33,36^ Furthermore, THG had very similar performance on the executive part as the CTG, providing evidence that the cognitive training was potentially responsible for these gains.

Comparing all groups after intervention, no significant differences were noted in the majority of cognitive variables. Hence, the results of this study indicate that no intervention was significantly superior to another, although each led to beneficial effects on sleep and cognition, indicating that the gains observed in the experimental groups were the result of the intervention sessions.

This study has some limitations, namely, the absence of objective measures to evaluate the gains associated with sleep and changes in sleep architecture; lack of control over the use of drugs, including hypnotics, as well as brain changes post- intervention in the structural and functional dimension. Furthermore, a small sample size may have limited the identification of the most significant differences between groups and possible generalizations for the population. Thus, future studies investigating the effects of cognitive training and/or sleep hygiene in the elderly should include objective measures of sleep and structural brain changes and employ longitudinal designs to verify the maintenance of gains post-intervention.

In summary, this study was consistent with the literature in showing cognitive training as an intervention to improve the cognitive performance of healthy elderly, not only for abilities directly trained during the sessions, but also for other functions, such as episodic memory. Moreover, the results also suggested that cognitive training has an impact on improving the quality of sleep and excessive daytime sleepiness.

Concomitantly, the intervention of sleep hygiene psychoeducation was also effective for promoting gains in some cognitive functions, especially episodic memory, planning and attention, as well as improved sleep quality and decreased sleepiness in the elderly people. However, combining cognitive training sessions with sleep hygiene psychoeducation failed to promote additional gains.
